# Bilateral simultaneous primary Oral Squamous Cell Carcinoma: A rare presentation

**DOI:** 10.1016/j.amsu.2022.104573

**Published:** 2022-09-08

**Authors:** Maira Adeel, Hira Andaleeb, Saad Shakil, Sareema Eman Akhtar, Talal Almas, Abdulla K. AlSubai, Sara AlNajdi, Aryam Mohammed Alenizi, Khaled Saeed Obaid Aldhaheri, Shakil Aqil

**Affiliations:** a Department of Otorhinolaryngology - Head and Neck Surgery, Liaquat National Hospital and Medical College, Pakistan; b Ziauddin Medical College, Karachi, Pakistan; c RCSI University of Medicine and Health Sciences, 123 St. Stephen's Green, Dublin 2, Ireland

**Keywords:** Oral carcinoma, Bilateral primary tumor, Squamous cell carcinoma, Simultaneous tumor, Bilateral buccal mucosa tumor, Bilateral simultaneous primary OSCC

## Abstract

**Introduction:**

and Importance: Oral Squamous Cell Carcinoma (OSSC) is one of the most common malignancies of the oral cavity and is one of the ten most prevalent tumours in the world. Control of this tumor is difficult and challenging as its propensity to spread is embedded in the cancer field of epithelial cells which alter these cells and induce a malignant potential within them. Simultaneous bilateral primary tumours are a rare presentation in the oral cavity which highlights the significance of early diagnosis and treatment.

**Case presentation:**

Here we present a case of 50-year-old gentleman known case of diabetes, hypertension and chronic history of beetle nut chewing who developed a simultaneously growing bilateral primary OSSC in the buccal mucosa. CT scan revealed a heterogeneous enhancing thickening in the bilateral buccogingival mucosa. A wide local excision of bilateral buccal mucosa with bilateral marginal mandibulectomy with neck dissection was planned.

**Clinical discussion:**

The majority of the case report emphasises the relevance of simultaneously developing bilateral primary oral cavity tumours in a patient who had a history of consuming beetle nuts. The independent incidence of bilateral primary OSSC in individuals without a history of tobacco, beetle nut, or alcohol use has also been documented in a small body of research. Due to the considerable clinical variety in its presentation, it is necessary to include bilateral primary OSSC when making a differential diagnosis of OSSC.

**Conclusion:**

Multiple bilateral primary tumours of the oral cavity are typically on the rise. The prognosis and survival of these individuals are considerably improved by close surveillance and early, expectant management of these cancers. This case study emphasises the value of thorough screening techniques used at an early stage to find these lesions and treat them appropriately.

## Highlights

• Oral Squamous Cell Carcinoma is one of the most prevalent malignancies in the world.
• The case presented is a bilaterally occurring simultaneous primary OSCC of the buccal mucosa in a 50-year-old patient.
• This is especially significant because bilateral primaries occurring concurrently are an uncommon occurrence in the oral cavity.
• Detecting and treating bilateral primaries timely is of utmost importance for better prognosis in view of surgical complications.

## 1 Introduction

Oral squamous cell carcinoma (OSCC) is the sixth most common cancer across the world [1]. Every year over 300,000 new cases of oral cancers are identified globally of which 90% are squamous cell carcinoma [2]. Multiple primary oral cancer (MPOC) most commonly involves the buccal mucosa followed by tongue and gingivae [3](4). According to Xiaoyu Lin, the worldwide mortality of oral cancer was 1.94/100,000. OSCC accounts for 8%–10% of all malignancies in Pakistan and India, accounting for one-third of the total cancer burden [4].(5).

Simultaneously growing tumours are the tumours that are diagnosed as primary tumor at the same time where as a tumor diagnosed within six months of primary tumor is a synchronously growing tumor or a secondary neoplasm. Another term for a tumor diagnosed within more than six months is a metachronous tumor [5].

## 2 Case report

**History and Examination:** A 50 years old male known case of diabetes and hypertension came with the complaint of bilateral buccal mucosa lesion for a period of 4 months. He was addicted to beetle nut chewing. The lesion was gradually increasing in size. It was mildly painful but relieved by taking painkillers. There was no history of cheek biting, dental trauma or dental procedures. There was no association of hoarseness, odynophagia, dysphagia, oral bleed or discharge from the lesion. However, it was associated with undocumented weight loss in a period of 4 months. There were no other ENT related complains. The patient presented with grade 3 trismus due to which the patient could not open his mouth and it was difficult to examine the tumor.

On examination of the oral cavity mouth opening was 2.5cm with an ulcerative lesion present on the right buccal mucosa starting from the first molar and involving the upper buccogingival sulcus reaching up to the lower buccogingival sulcus (Fig. 5). Posteriorly it was involving the retro molar trigone. There was another lesion on the left side of the buccal mucosa starting from the second molar posteriorly involving the retro molar trigone (Fig. 6). Superiorly involving the upper buccogingival sulcus and upper alveolar mucosa and reaching up to the lower buccogingival sulcus. There were multiple lymph nodes palpable from level 1 to 3 on the left side. Largest one approximately 2.5*3.0cm, firm and mobile at left level 1b. He was advised for a biopsy under local anaesthesia. Frozen specimen (Fig. 7) was taken and sent for investigation.

**Fig. 1:**
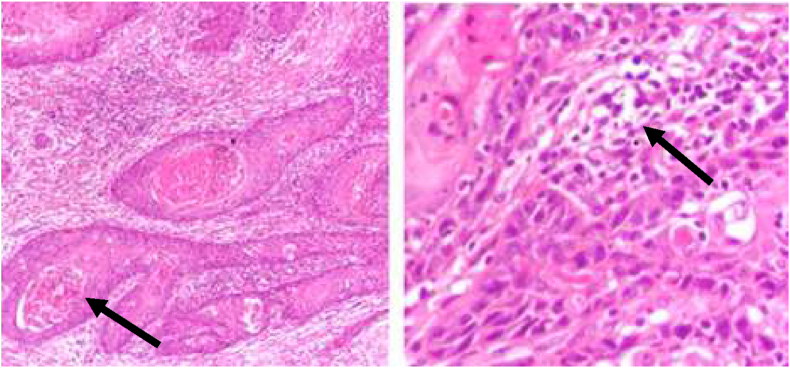
Right buccal mucosa histology.

**Fig. 2:**
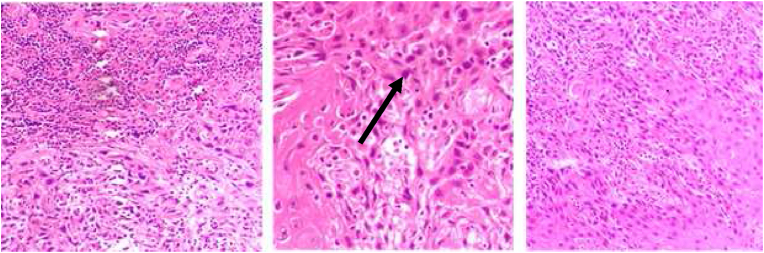
Left buccal mucosa histology.

**Histopathology**: An incisional biopsy was taken from bilateral lesions which came out to be well to moderately differentiated squamous cell carcinoma (Fig. 1, Fig. 2).

**Fig. 3:**
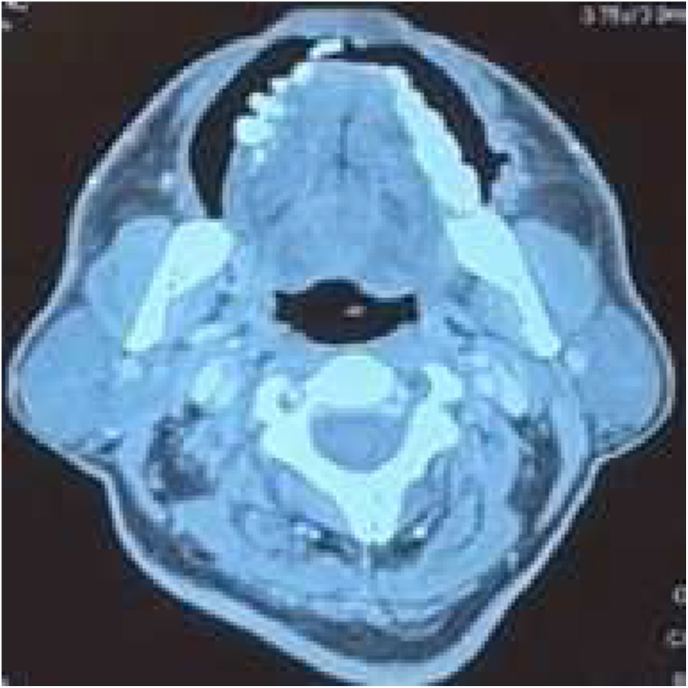
Right buccal mucosa CT scan.

**Fig. 4:**
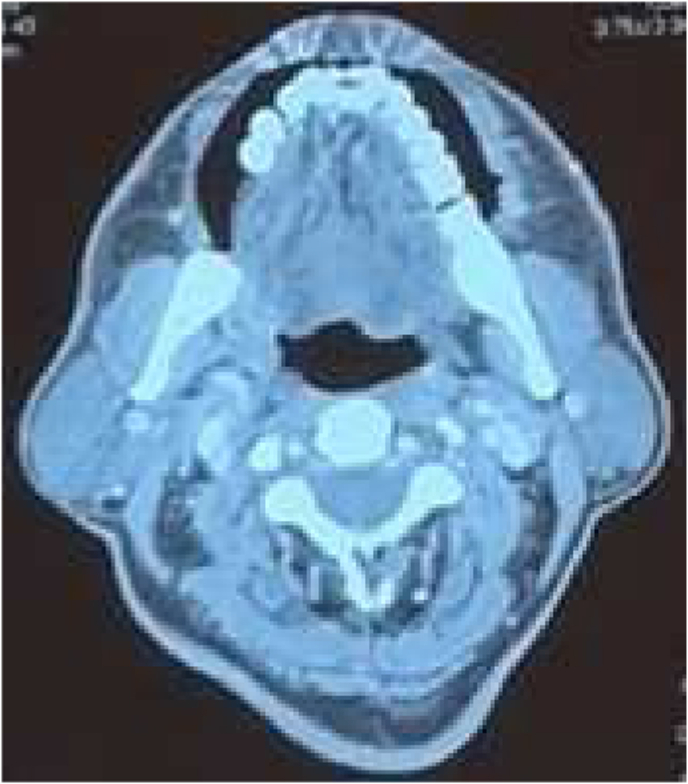
Left buccal mucosa CT scan.

**Radiological Findings:** CT scan neck and chest with contrast was done which showed a heterogeneous enhancing thickening in the bilateral buccogingival mucosa (Fig. 3 and Fig. 4). Pre op staging according to NCCN guidelines was T1N2cMo – stage 4A.

**Fig. 5:**
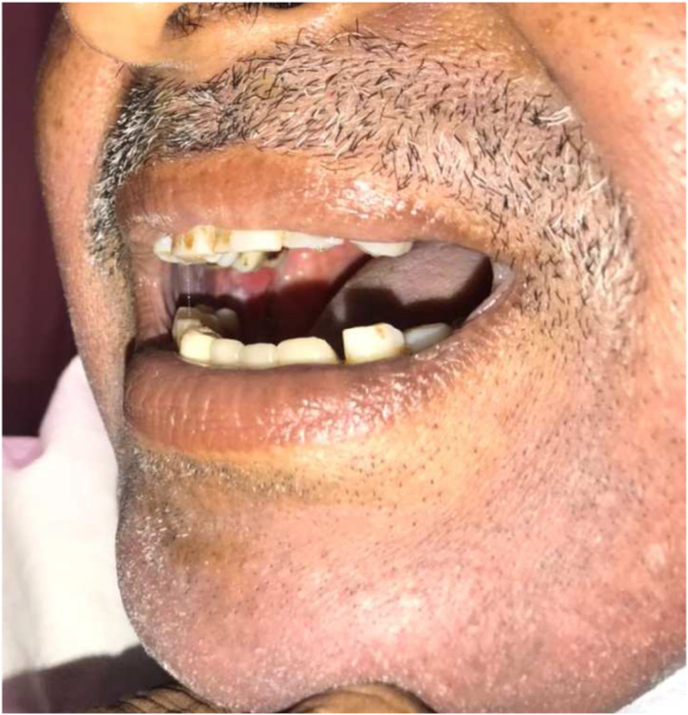
Right buccal mucosa pre-operative.

**Fig. 6:**
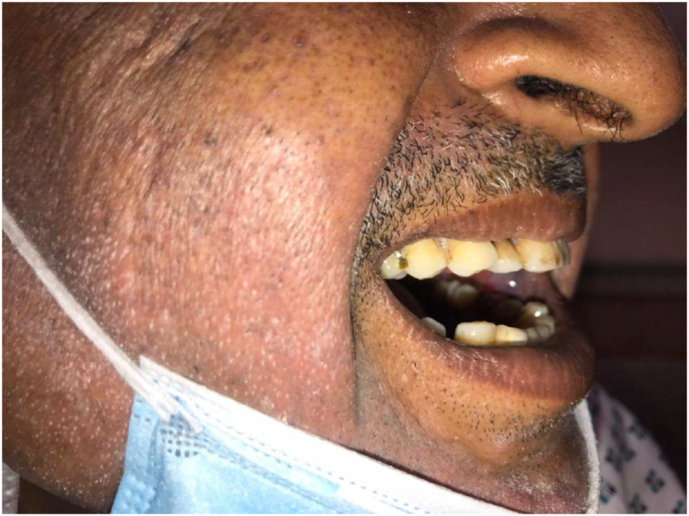
Left buccal mucosa pre-operative.

**Fig. 7:**
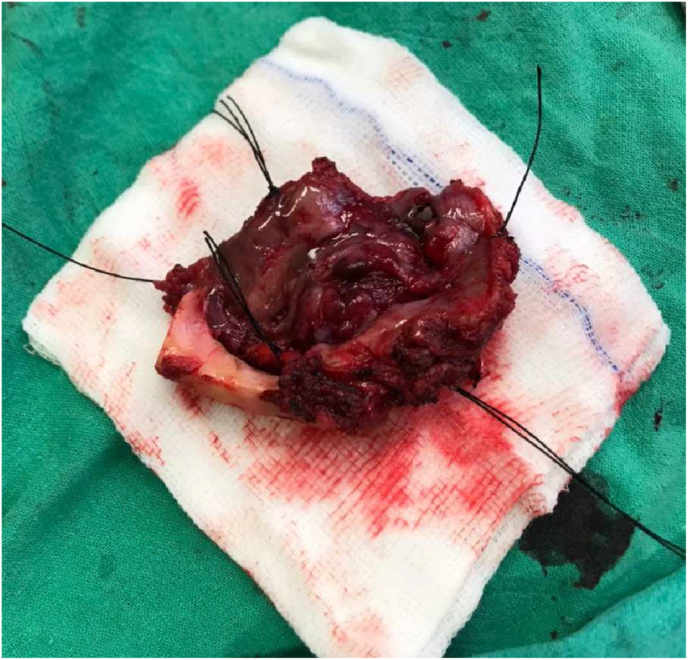
Frozen specimen.

**Fig. 8:**
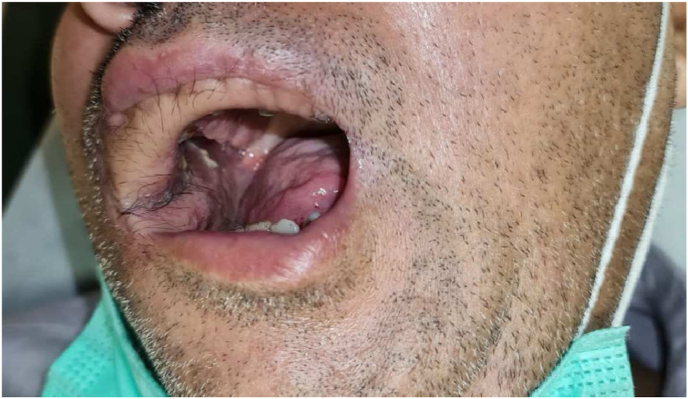
Reconstruction after wide excision.

A wide local excision of bilateral buccal mucosa lesion was planned along with bilateral marginal mandibulectomy combined with left modified radical neck dissection (type 2), right selective neck dissection level 1 to 4, tracheostomy and reconstruction with free flap (Fig. 8). The present paper has been reported in line with the SCARE 2020 criteria [6].

## 3 Discussion

Squamous cell carcinoma is one of the most prevalent oral cancers, with an average survival rate of approximately five years [6]. Multiple primaries in the head and neck region are known to affect 7%–21% of the population, with 1.4% of them restricted to the oral cavity. Currently there is little published literature on multiple primaries of the oral cavity despite the fact it appears to be on the rise. This emphasises on the significance of this case presented. A similar case of simultaneously growing squamous cell carcinoma was also reported by Camila and et al., in 2020 where two lesions were simultaneously growing one in the palate and the other on the alveolar ridge [7].

Buccal mucosa and vestibule are most commonly associated with OSCC and have multiple risk factors including the long-term usage of tobacco (45%), beetle nut (14%), areca nut (25%) and alcohol (16%) [8]. Simultaneous tumours of the oral cavity and hypopharynx has a incidence ranging from 1% to 7.4% as reported by 6 separate studies [9]. Presence of multiple primary tumours doesn't affect the overall survival as compared to the single primary cancer. In a study conducted by Ya-Dong Li et all it was reported that the disease specific survival rate for multiple primary tumours was reported to be 90.7% and 79.6% at 5 years and 10 years respectively [10].

The concept of field cancerization is applied in the case of simultaneous/multiple primary tumours in oral cavity where a wide area of anaplasia and mucosal change secondary to intake of chewing tobacco, involving multiple sites and leading to areas of skip lesions contributing to premalignancy [6]. The following criteria must be met in order to diagnose multiple primary tumours: I each tumor must be unique and structurally different. When dysplastic mucosa is found nearby, a multi-centric primary neoplasm is suspected; ii) It's important to rule out the possibility of a second primary carcinoma that is a metastasis or a local recurrence. It must develop 3 years after the first diagnosis or be at least 2 cm away from the original tumor on the normal epithelium.

Previously there have been reports of synchronous and metachronous lesions however simultaneous bilateral primaries have rarely been reported. Hence this case report is vital for ENT and head and neck surgeons for effective and efficient timely diagnosis and the required treatment.

## 4 Conclusion

With appropriate imaging modality is essential in making the diagnosis of bilateral simultaneous primary tumours of oral cavity. Wide local excision with good clearance margins (0.5cm–1cm) is the key to achieve good clearance. The overall survival of multiple primary tumours is not different from single primary tumor and its prognostic factors are also similar. This study focuses in particular on the variations in oral tumor presentation. If there is bilateral involvement of the oral tumor and neck lymph nodes, surgery may be challenging, and the prognosis is poor. Therefore, early detection of the local and regional spread is crucial.

## Ethical approval

NA.

## Sources of funding

None to declare.

## Author contribution

Maira Adeel: Study design and writing of case report body. Also reviewed the article, Hira Andaleeb: Study design and reviewed the article, Saad Shakil: Literature search. Writing of manuscript introduction and discussion, Sareema Eman Akhtar: Literature search. Writing of manuscript introduction and discussion, Talal Almas: Supervised and reviewed the article, Abdulla K. AlSubai: Supervised and reviewed the article, Sara AlNajdi: Supervised and reviewed the article, Aryam Mohammed Alenizi, **Khaled Saeed Obaid Aldhaheri**: Supervised and reviewed the article, Shakil Aqil: Supervised and reviewed the article. The manuscript has been read and approved by all authors, requirements of authorships have been met and each author believes that the manuscript represents honest work.

## Registration of research studies

Name of the registry: NA
Unique Identifying number or registration ID: NA
Hyperlink to your specific registration (must be publicly accessible and will be checked): NA

## Guarantor

Talal Almas RCSI University of Medicine and Health Sciences 123 St. Stephen's Green Dublin 2, Ireland Talalalmas.almas@gmail.com.

## Consent

Written informed consent was obtained from the patient for publication of this case report and accompanying images. A copy of the written consent is available for review by the Editor-in-Chief of this journal on request.

## Provenance and peer-review

Not commissioned, externally peer-reviewed.

## Declaration of competing interest

None to declare.
